# A Biosensor for Simultaneous Detection of Epinephrine and Ascorbic Acid Based on Fe(III)–Polyhistidine-Functionalized Multi-Wall Carbon Nanotube Composites

**DOI:** 10.3390/ijms25147883

**Published:** 2024-07-18

**Authors:** Bingkai Han, Yuan Chen, Hongtao Wang, Jilong Yan, Guang Liu, Ziru Huang, Chenghang Zhou

**Affiliations:** 1 Tianjin Key Laboratory of Exercise Physiology and Sports Medicine, Institute of Sport, Exercise & Health, Tianjin University of Sport, No. 16 Donghai Road, West Tuanbo New Town, Jinghai District, Tianjin 301617, China; 2 The Key Laboratory of Bioactive Materials, Ministry of Education, College of Life Science, Nankai University, Weijin Road No. 94, Tianjin 300071, China; 3 College of Life Science, Henan University, No. 379 Mingli Road, Kaifeng 475004, China

**Keywords:** epinephrine, ascorbic acid, polyhistidine, multi-wall carbon nanotube, biosensor

## Abstract

Epinephrine (EP) is a very important chemical transmitter in the transmission of nerve impulses in the central nervous system of mammals. Ascorbic acid (AA) is considered to be the most important extracellular fluid antioxidant and has important antioxidant properties in the cell. In this study, a series of transition metal–polyhistidine-carboxylated multi-wall carbon nanotube nanocomposites were synthesized, and their simultaneous catalytic effects on epinephrine and ascorbic acid were investigated. The results showed that nanocomposites based on iron ions had the highest catalytic activity. The prepared biosensor expressed high selectivity toward EP and AA with LOD values of 0.1 μΜ (AA) and 0.01 μΜ (EP), and sensitivity values of 4.18 μA mM^−1^ with a range of 0.001–5 mM (AA), 50.98 μA mM^−1^ with a range of 0.2–100 μM (EP), and 265.75 μA mM^−1^ with a range of 0.1–1.0 mM (EP). Moreover, it showed good stability, good repeatability and high selectivity in real sample detection. This work is a reference for the design of new electrochemical enzyme-free biosensors and the detection of biomarkers.

## 1. Introduction

Epinephrine (EP) is a hormone and neurotransmitter synthesized and released by chrome cells in the adrenal medulla. It is an extremely important chemical transmitter in the central nervous system of mammals for transmitting nerve impulses and is nicknamed the “fight or flight” hormone. It is released into the blood system in response to excitement, fear, anger and other stimuli to raise blood sugar levels. Clinically, epinephrine is often used to treat cardiac arrest, anaphylactic shock and bronchial asthma [1,2]. Studies have shown that epinephrine can induce the apoptosis of cardiomyocytes, nerve cells and coronary endothelial cells by regulating FAS-FASL-mediated apoptosis and activating the caspase-3 signaling pathway under pathological conditions [3]. On the other hand, epinephrine can improve the level of basic metabolism as well as promote glycolysis, protein decomposition and fatty acid oxidation, which can trigger the body’s oxidative stress response under special conditions, leading to the occurrence and development of certain diseases, and chemical transmitters such as EP can activate certain tumor adrenergic receptor-related signal transduction pathways. It plays a certain role in the process of tumor occurrence and development [4].

Oxidative stress has long been believed to be a negative effect caused by free radicals in the body and is related to aging and diseases. There are two kinds of antioxidant systems in response to oxidative stress: one is the enzyme antioxidant system, which includes catalase and superoxide dismutase; the other is a type of non-enzymatic antioxidant system that mainly includes reducing some physiologically active substances, such as ascorbic acid (vitamin C, L-ascorbic acid, AA), vitamin E and glutathione. Among many antioxidant substances, ascorbic acid is considered to be the most important extracellular liquid antioxidant and also has important antioxidant properties in the cell, which can effectively remove OH∙, H_2_O_2_, etc. When the human body is exposed to an environment that can cause oxidative damage, such as excessive fatigue, ultraviolet radiation, and smoking, ascorbic acid becomes the first line of defense of antioxidants in the blood [5,6].

Both epinephrine and ascorbic acid have strong reducibility, but they play diametrically opposite antagonistic roles in the body’s oxidative stress response. Adrenalin acts as a signaling molecule that produces free radicals by promoting basal metabolic and aerobic processes; ascorbic acid acts as a basic antioxidant, constantly monitoring the production of excessive free radicals in order to clean up in time. For these two substances, a fast, convenient, cheap and efficient detection technology is of great significance in both basic research and clinical diagnosis.

The detection methods for epinephrine and ascorbic acid usually include high-performance liquid chromatography [7], mass spectrometry [8] and fluorescence spectrometry [9]. These conventional detection methods have the disadvantages of expensive equipment, complicated operation, and specialized sample processing. In today’s rapidly developing society, it is increasingly difficult to meet daily needs; therefore, a fast, cheap and efficient analysis and detection system is urgently required. As a new instrument and technical means, biosensors are attracting increasing attention. Epinephrine and ascorbic acid are generally present in biological samples at the same time. However, the oxidation potential of ascorbic acid on the surface of most solid electrodes is very close to that of epinephrine, which is prone to overlapping responses, so it is difficult to achieve the simultaneous detection of both. The usual solution is to eliminate ascorbic acid’s interference with epinephrine [10] or to separate the chemical reactions [11,12]. Hu et al. constructed a simple electrochemical sensor by modifying carbon nanotubes on the surface of glassy carbon electrodes. They used cyclic voltammetry to simultaneously detect epinephrine and ascorbic acid, and they observed the oxidation potential of negatively shifted ascorbic acid, thus realizing an effective distinction between the two [13]. Sun et al. constructed a simple electrochemical sensor by synthesizing a layer of benzotriazole self-assembly film on the surface of a gold electrode, which effectively distinguished the oxidation potential of epinephrine and ascorbate, and the oxidation peak potential difference between the two was 200 ± 10 mV, thus realizing the simultaneous detection of both [14].

Since Japanese scientist Iijima [15] discovered carbon nanomaterials with a coaxial tubular structure—carbon nanotubes in 1991, carbon nanotubes (CNTs) have been characterized by their special nanostructures, excellent mechanical properties and electrical conductivity. They have been extensively researched and applied as biosensors [16,17], catalytic materials [18], energy storage materials [19], drug carriers [20], etc. Carbon nanotubes have excellent electrical conductivity, and their electron transfer efficiency is affected by the length of carbon nanotubes to a certain extent, and electron transfer is mainly carried out along the radial direction of carbon nanotubes rather than through the diameter of the tube [21]. Compared with randomly distributed carbon nanotubes, carbon nanotubes microarrays, or aligned carbon nanotubes, it is possible to effectively avoid overlapping entanglement between carbon tubes due to their highly ordered parallel arrangement structure and reduced contact resistance [22]. Therefore, short carbon nanotubes with an ordered structure have better electrical conductivity. Carboxylated multi-walled carbon nanotubes (CMWCNTs) arrays cut short and open by chemical oxidation have significant advantages in improving electron transport efficiency. In addition to the above highly ordered structures, the inner wall of CMWCNTs also participated in electron transport and had a large current-carrying capacity [23]. At the same time, CMWCNTs also play a crucial role in improving the performance of biosensors.

Poly-L-histidine (PLH) is a multifunctional polyelectrolyte that can be used to modify CMWCNTs microarrays. PLH molecules contain the lipophilic π–π-conjugated aromatic functional group imidazolyl, so PLH can achieve non-covalent bonding with CMWCNTs through π–π stacking. At the same time, PLH can also achieve covalent cross-linking through the formation of amide bonds between its free amino group and the carboxyl group of CMWCNTs. Both π–π-conjugated electrons and lone pairs play an important role in the electron transport process [24,25]. The imidazole group in the PLH side chain acts as a ligand that can chelate a variety of metal ions or atoms, and it can also achieve direct electron transfer to CMWCNTs. Therefore, the modification of PLH on the surface of CMWCNTs arrays can significantly enhance electron transport. In addition, it has been confirmed that metal ions (or atoms)–polyhistidine complexes have obvious catalytic effects on many substances.

In this study, a series of transition metal–polyhistidine-carboxylated multi-wall carbon nanotube nanocomposites were synthesized by chelating polyhistidine–imidazolyl to transition metal ions, and their simultaneous catalytic effects on epinephrine and ascorbic acid were investigated. The results showed that these nanocomposites could distinguish the oxidation potential of epinephrine and ascorbic acid effectively, and the nanocomposites based on iron ions had the highest catalytic activity. Therefore, in this study, nanocomposite materials based on iron ions were selected for the construction of a biosensing interface and the simultaneous detection of epinephrine and ascorbic acid.

## 2. Results and Discussion

### 2.1. Screening of Transition Metal Ions in Me-PLH-CMWCNTs Nanocomposites

**Figure 1:**
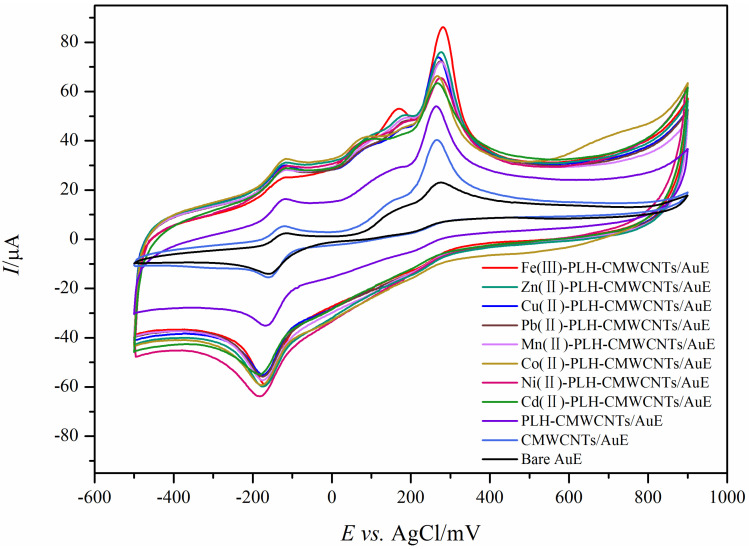
Cyclic voltammograms of 1 mM AA and 0.5 mM EP at a series of electrodes with different modifications. Scan rate: 50 mV s^−1^, scan area: −500~800 mV.

In this study, cyclic voltammetry was used to investigate the electrocatalytic effects of a series of nanocomposite electrodes based on transition metal ions on epinephrine and ascorbic acid at the same time. The results are shown in Figure 1. On the surface of the bare gold electrode, both EP and AA showed characteristic REDOX peaks with a large EP oxidation peak near 250 mV and a pair of smaller REDOX peaks around −180 mV. The oxidation peak of AA appears near 100 mV. Due to the small difference in oxidation potential between the two, and the low response current of AA on the bare electrode surface, it is not easy to distinguish the interference from EP. The response of gold electrodes modified with CMWCNTs and PLH-CMWCNTs to EP and AA was significantly enhanced. According to the analysis described in the previous section, this was due to the increased electron transfer rate and specific surface area at the electrode interface promoted by CMWCNTs and PLH-CMWCNTs. The response of nanocomposites containing different transition metal ions to EP and AA was significantly improved, indicating that transition metal ions play an obvious role as catalysts in the electrocatalytic oxidation of the two substances. Among them, the effect of iron ion is the most significant.

**Figure 2:**
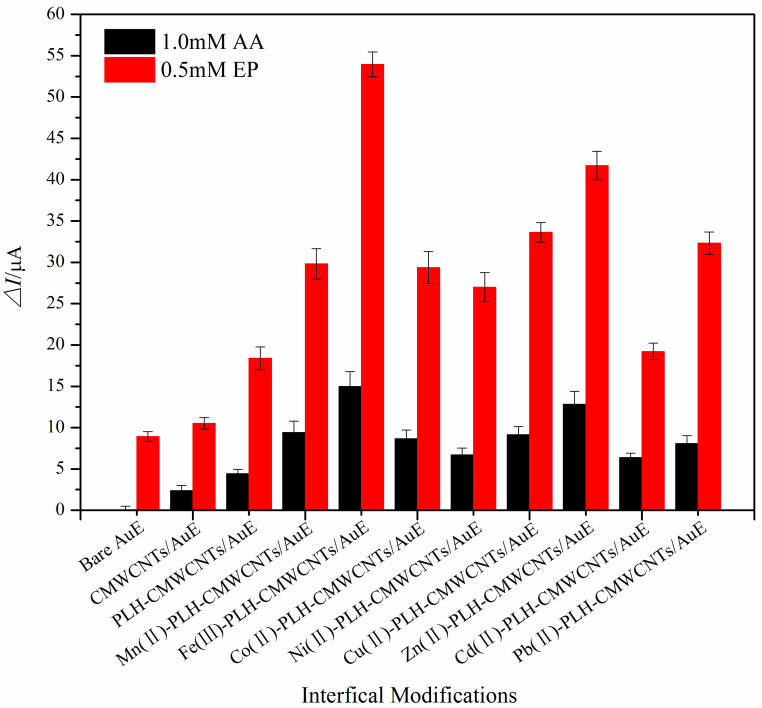
Comparison of the oxide peak currents of a series of electrodes with different modifications toward 1 mM AA and 0.5 mM EP via differential pulse voltammetry.

In order to select the ion with the best catalytic effect, the electrocatalytic effect of the nanocomposite electrode based on the transition metal ion on EP and AA was investigated using the differential pulse method. The catalytic effect of this method is basically the same as that of cyclic voltammetry, in which the bare gold electrode has almost no response to AA, EP only has an oxidation peak near 150 mV, AA has an oxidation peak near −50 mV, and the oxidation potential difference between the two is larger and easier to distinguish than that of cyclic voltammetry. Figure 2 presents the oxidation peak current of different modified electrodes. It can be seen that among the eight transition metal ions, iron ion has the best catalytic effect. Therefore, iron ion was selected as the catalytic active center ion for the simultaneous detection of EP and AA in this study.

### 2.2. Morphological Characterization of Fe(III)-PLH-CMWCNTs

**Figure 3:**
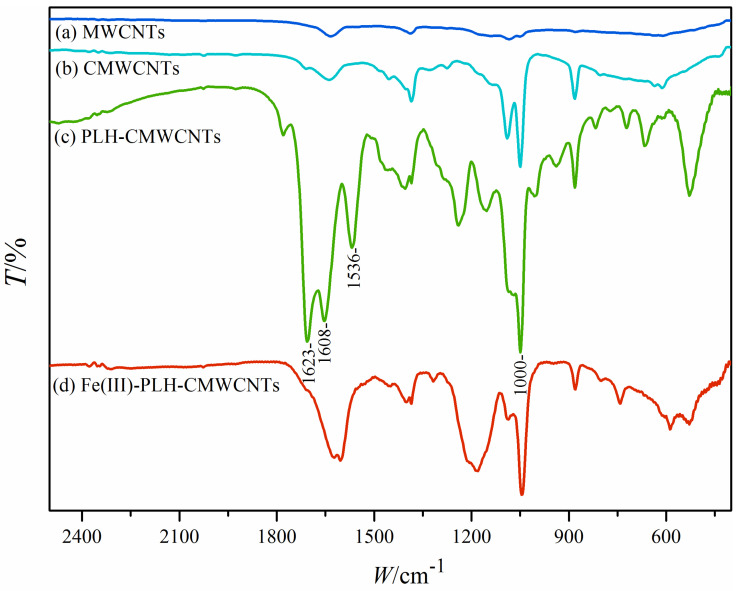
FTIR spectra of MWCNTs (**a**), CMWCNTs (**b**), PLH-CMWCNTs (**c**) and Fe(III)-PLH-CMWCNTs (**d**).

Firstly, the composite materials were characterized and analyzed. Figure 3 shows the FTIR spectra of each nanomaterial, in which the stretching vibration of carboxyl group is represented at 1000 cm^−1^. Compared with curve a and curve b, it can be seen that the carboxyl absorption peak of CMWCNTs is significantly higher than that of MWCNTs, indicating that CMWCNTs with a good carboxylation effect were obtained in this experiment. In curve c, 1623 cm^−1^, 1608 cm^−1^ and 1536 cm^−1^ represent the stretching vibrations of C=O, C=N and C-N, respectively. Compared with curve b, the increase in absorption peaks at these positions is mainly attributed to the modification of PLH, which also indicates that PLH has been successfully modified to CMWCNTs. The peaks of the above characteristic peaks in curve d are significantly lower than those in curve c. The reason may be that the modification of iron ions interferes with the absorption of infrared light by these groups, which also indicates that iron ions have been chelated into the nanocomposites.

**Figure 4:**
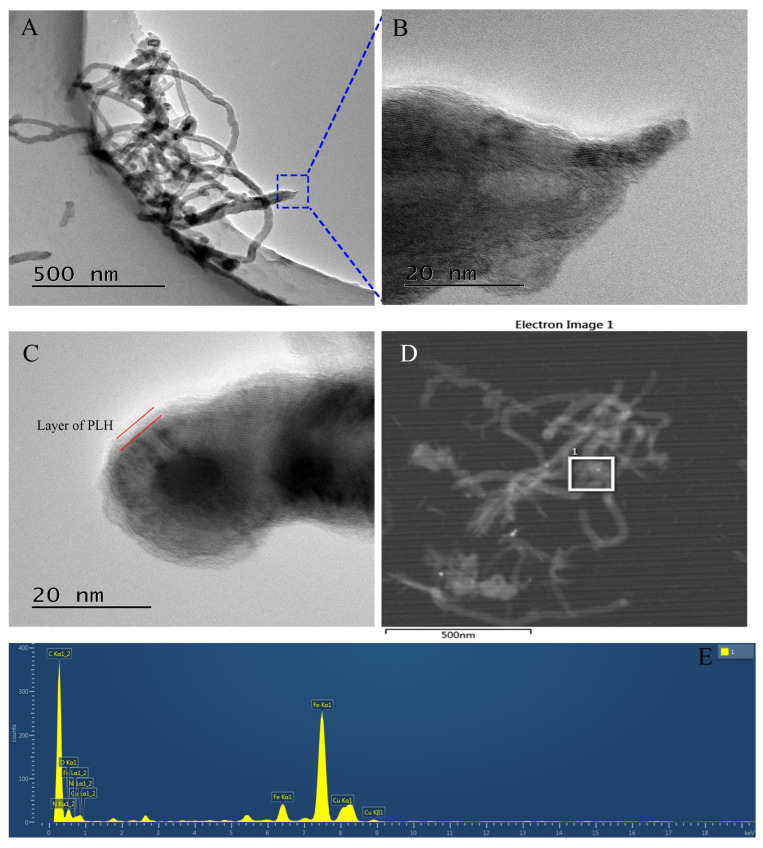
TEM images of MWCNTs (**A**), CMWCNTs (**B**), PLH-CMWCNTs (**C**), HADDF image (**D**) at different magnifications and EDX spectrum (**E**) of Fe(III)-PLH-CMWCNTs. Materials were dispersed by ultrasound, and the samples were made of 3 mm (200 mesh) copper mesh with a microgrid carbon film.

Figure 4A shows the TEM image of CMWCNTs, in which the typical hollow tubular structure of multi-walled carbon nanotubes and some carbon nanotubes cut short by mixed acid oxidation can be observed. Figure 4B shows the tube end enlargement of one CWMCNTs segment. The multilayer graphite lamellar structure on the wall of the carbon nanotube and the opening of the tube end truncated by carboxylation can be clearly observed. Figure 4C shows a typical PLH-modified CMWCNTs with closed tube ends, from which a PLH film about 2 nm thick on the outer side of the tube wall can be clearly observed. Figure 4D shows the local high-angle toroidal dark field (HADDF) image of Fe(III)-PLH-CMWCNTs. EDX spectral analysis was performed on the region in the white box. The results are shown in Figure 4E. Peak C in the spectrum came from CMWCNTs and PLH, while peak N came from PLH. The elemental composition (wt%) of the Fe(III)-PLH-CMWCNTs shows 83.62% (C), 1.12% (N), 3.55% (O), 8.48% (Fe) and 3.23%(Cu). This indicates that iron ions have been successfully chelated into the nanomaterial, and the Cu peak in the figure is mainly from the copper microgate supporting film prepared by TEM.

Also, the X-ray photoelectron spectroscopy (XPS) of the Fe(III)-PLH-MWCNTs nanocomposites shows that the C 1s, N 1s O 1s, Fe 2p_1/2_ and Fe 2p_3/2_ regions are at 283.8 eV, 399.1 eV, 530.1 eV, 724.7 eV, and 711.1 eV (Figure S1A), respectively, which can be clearly observed, verifying the presence of C, O, Fe, and N elements in Fe(III)-PLH-MWCNTs. C–C (284.8 eV), C–N (286.5 eV), and O–C=O (288.8 eV) confirm the compound of PLH and the carboxylation of MWCNTs (Figure S1B). The deconvolution of Fe 2p_3/2_ coincided with the tetrahedral and octahedral site of Fe, verifying there was Fe^3+^ in Fe(III)-PLH-MWCNTs.

Combining FTIR, TEM, XPS and EDX characterization, it is fully proved that Fe(III)-PLH-CMWCNTs nanocomposites have been successfully prepared in this study.

### 2.3. Electrochemical Activity of Fe(III)-PLH-CMWCNTs/AuE

**Figure 5:**
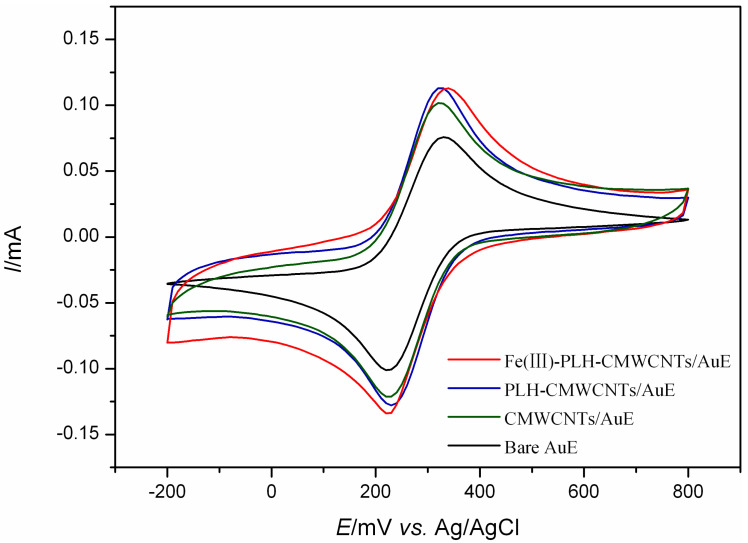
Cyclic voltammograms of 10 mM K_3_[Fe(CN)_6_] at bare AuE, CMWCNTs/AuE, PLH-CMWCNTs/AuE and Fe(III)-PLH-CMWCNTs/AuE recorded in 0.1 M KCl aqueous. Scan rate: 50 mV s^−1^, scan area: −200~800 mV.

The electrical conductivity of nanocomposites and the effective active area of gold electrodes modified with different materials were analyzed with cyclic voltammetry in a potassium ferricyanide system, and the results are shown in Figure 5. The electrodes modified with different materials showed a characteristic REDOX peak of [Fe(CN)_6_]^4−/3−^ in the potassium ferricyanide system, and the REDOX current of the gold electrode modified with CMWCNTs and PLH-CMWCNTs was significantly increased, indicating that the electrodes modified with CMWCNTs had good conductivity and a large specific surface area. PLH-CMWCNTs competitively bind iron ions in [Fe(CN)_6_]^4−/3−^ due to the chelation of PLH side-chain imidazolyl. Therefore, although Fe ions are modified in Fe(III)-PLH-CMWCNTs, the conductivity of the potassium ferricyanide system does not improve. The relative surface area of the modified electrode is similar to that of the PLH-CMWCNT-modified electrode. The effective active area of different modified electrodes can be obtained using the Randles–Ševčík Equation (1) [26]:

(1) $$i_{p}=2.69\times 10^{5}n^{3/2}D^{1/2}C_{0}v^{1/2}A_{eff}$$

where $i_{p}$ stands for the peak current (μA); *n* represents the number of electron transfers in the REDOX reaction, which is 1; D represents the molecular diffusion coefficient in the solution, which is (6.70 ± 0.02) × 10^−6^ cm^2^ s^−1^; *C*_0_ represents the concentration of probe molecules in the solution, that is, 10 mM; *v* represents the scanning rate, which is now 50 mV s^−1^; and A*_eff_* corresponds to the effective active area (cm^2^) of the modified electrode. The effective active area of different modified electrodes can be obtained by substituting the peak oxidation current of each electrode in Figure 5 into the above equation. Among them, the effective active area of Fe(III)-PLH-CMWCNTs/AuE was 0.113 cm^2^, which is the same as that of PLH-CMWCNTs/AuE, and it is 1.6 and 1.2 times that of CMWCNTs/AuE and bare gold electrodes, respectively. We have reason to believe that it is the introduction of polyhistidine that significantly improves the electrical conductivity of the metal–organic nanocomposite and also determines the high sensitivity of the sensor constructed in this study.

**Figure 6:**
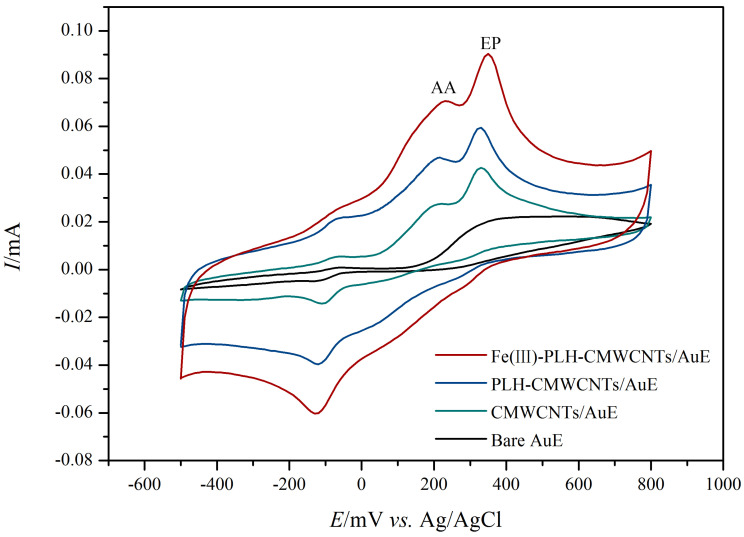
Cyclic voltammograms of 1 mM AA and 0.5 mM EP at bare AuE, CMWCNTs/AuE, PLH-CMWCNTs/AuE and Fe(III)-PLH-CMWCNTs/AuE recorded in 0.1 M PBS (pH 7.0). Scan rate: 50 mV s^−1^, scan area: −500~800 mV.

The electrocatalytic effects of Fe(III)-PLH-CMWCNTs/AuE on 1.0 mM AA and 0.5 mM EP in a 0.1 M PBS (pH 7.0) system were investigated using cyclic voltammetry. As shown in Figure 6, the electrocatalytic oxidation behaviors of AA and EP on different modified electrode surfaces are basically consistent with those discussed before. It was also observed that in the response of the bare gold electrode to AA and EP at this time, it is impossible to distinguish the oxidation peak of the two, mainly because on the surface of the bare gold electrode, EP is prone to electropolymerization, so the electrode passivation, which also shows the necessity of using nanocomposite materials to modify the electrode, on the one hand, can effectively prevent EP electropolymerization, avoiding the loss of sensitivity of electrode passivation. On the other hand, it can effectively distinguish the oxidation potential of AA and EP and improve the catalytic effect.

**Figure 7:**
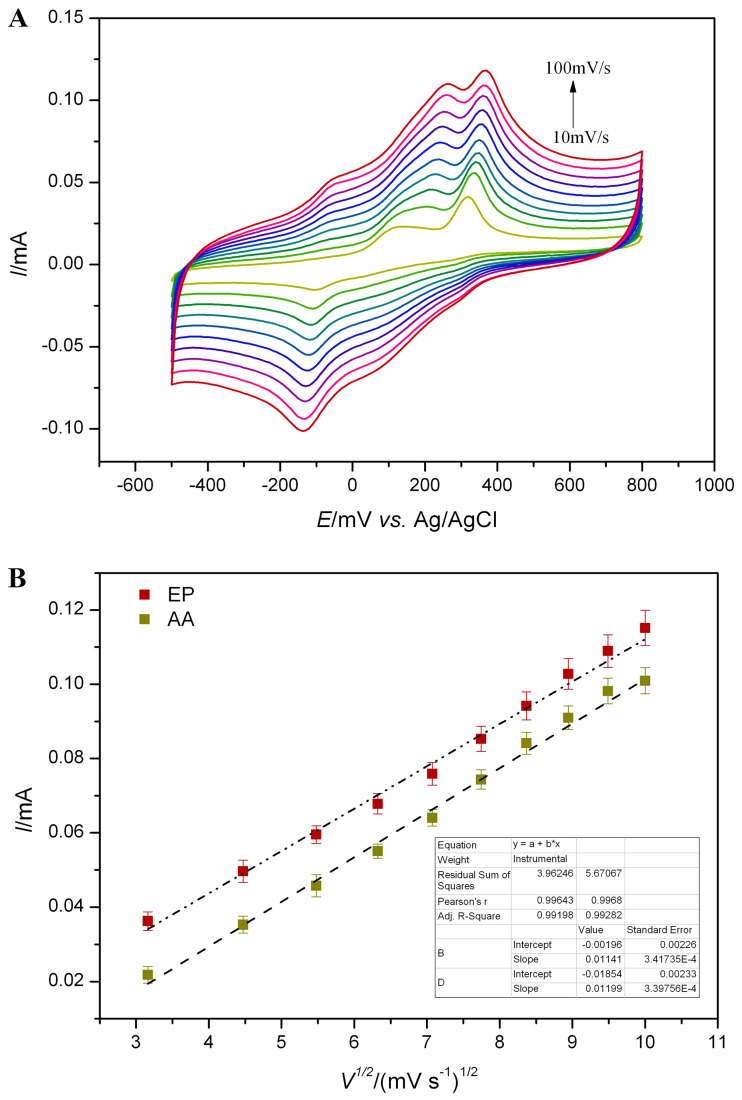
(**A**) Cyclic voltammograms of 2 mM AA and 0.4 mM EP at Fe(III)-PLH-CMWCNTs/AuE under the scan rate ranged from 10 to 100 mV s^−1^, scan area: −500~800 mV. (**B**) The linear fitting curves of oxidation peak currents of AA and EP.

The electrocatalytic behavior of Fe(III)-PLH-CMWCNTs/AuE on 1.0 mM AA and 0.5 mM EP at different scanning rates was also investigated, and the results are shown in Figure 7. It can be seen from Figure 7A that the oxidation currents of both AA and EP gradually increase with the increase in scanning rate. The peak oxidation current of AA and EP (at 250 mV) is linearly fitted to the square root of the scanning rate, and it is found that it presents a good linear relationship. The linear equations for AA and EP are Ip(AA) = 0.01199C (AA) − 0.01854 (R^2^ = 0.993) and Ip(EP) = 0.01141C (EP) − 0.00196 (R^2^ = 0.992), respectively (as shown in Figure 7B). It is shown that the electrocatalytic oxidation of AA and EP at the sensor interface constructed in this study is a diffusion-controlled process. The slopes of the two were substituted into Equation (1), and according to the effective active area of Fe(III)-PLH-CMWCNTs/AuE (0.113 cm^2^), the diffusion coefficient of AA was 1.94 × 10^−5^ cm^2^ s^−1^ and that of EP was 1.76 × 10^−5^ cm^2^ s^−1^. These values are basically consistent with those previously reported (5.9 × 10^−5^ cm^2^ s^−1^ [27], 1.24 × 10^−5^ cm^2^ s^−1^ [28]).

### 2.4. Simultaneous Detection of AA and EP by Fe(III)-PLH-CMWCNTs/AuE

**Figure 8:**
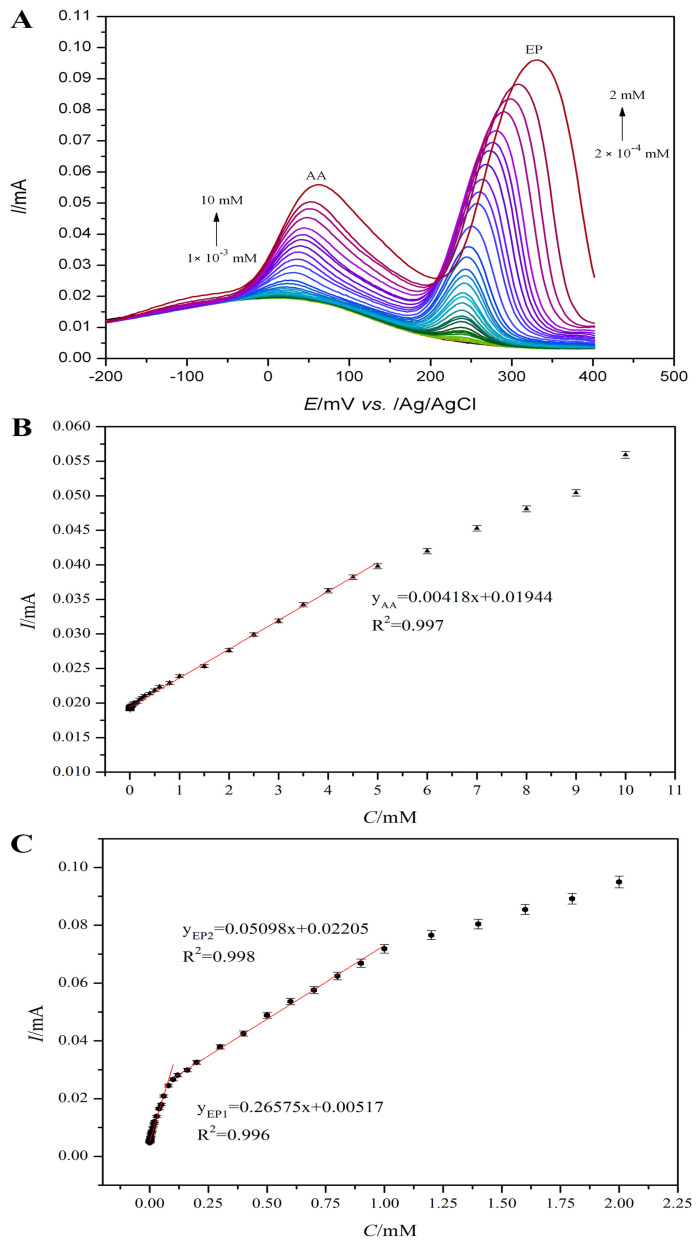
(**A**) Differential pulse voltammograms of AA and EP at the Fe(III)-PLH-CMWCNTs/AuE in series of concentrations. Scan area: −200~400 mV. (**B**) The linear relationship between response current and AA concentration. (**C**) The linear relationship between response current and EP concentration.

In this experiment, the simultaneous detection effect of Fe(III)-PLH-CMWCNTs/AuE on AA and EP in a 0.1 M PBS (pH 6.0) system was investigated using the differential pulse method, and the results are shown in Figure 8A. The oxidation potential of AA is about 50 mV, and the oxidation potential of EP is about 250 mV. At the same concentration, the oxidation current of EP is obviously higher than that of AA, but the oxidation potential of EP shifts slightly to the right with the increase in the concentration of AA and EP, which may be caused by the slight change in pH value of the solution caused by the increase in the concentration of AA and EP. The peak oxidation current and concentration of the two were linearly fitted, and the results are shown in Figure 8B,C. In the range of 0.001~5 mM, the oxidation current of AA showed a good linear relationship with the concentration. The regression equation was y_AA_ (mA) = 0.00418x (mM) + 0.01944 (R^2^ = 0.997), the sensitivity was 4.18 μA mM^−1^, and the lowest detection limit was 0.1 μm (S/N = 3). The linear regression equation is y_EP1_ (mA) = 0.26575x (mM) + 0.00517 (R^2^ = 0.996), and the sensitivity in this range is 265.75 μA mM^−1^. In the concentration range of 0.1~1.0 mM, the linear regression equation is y_EP2_ (mA) = 0.05098x (mM) + 0.02205 (R^2^ = 0.998), the sensitivity in this range is 50.98 μA mM^−1^ and the sensor’s lowest detection limit for EP is 0.01 μm (S/N = 3).

Table 1 compares the effects of Fe(III)-PLH-CMWCNTs/AuE and the previously reported sensors on the simultaneous detection of AA and EP. Compared with the minimum detection limit, detection range and sensitivity of each sensor, the biosensor based on iron (III)–polyhistidine-functionalized multi-wall carbon nanotube nanocomposite materials constructed in this study showed a higher sensitivity, wider detection range and lower detection limit than most reported sensors.

In summary, the biosensor constructed in this study showed excellent performance in terms of catalytic activity and sensitivity, which was mainly attributed to the high-efficiency electrocatalytic activity of Fe(III)-PLH in the metal–organic nanocomposite as the catalytic activity center. At the same time, PLH-CMWCNTs greatly improved the electron transfer efficiency at the biosensing interface, increased the effective active area, and ensured the enrichment of a large number of substances to be measured at the biosensing interface.

### 2.5. Reproducibility, Stability, Anti-Interference and Suitability Evaluation

Reproducibility is an important measure of a standard analytical method. The reproducibility of this study protocol was evaluated by using the same experimental procedure as above. Seven electrodes were prepared successively, and their electrochemical responses to 1.0 mM AA and 0.2 mM EP were investigated in a 0.1 M PBS (pH 6.0) system. The seven electrodes showed almost the same electrochemical response to AA and EP, and the standard deviation was 4.17%, indicating that the scheme had good reproducibility. The stability of the sensor constructed in this study was evaluated by examining the electrochemical response of 1.0 mM AA and 0.2 mM EP with the same electrode at an interval of 15 days. The results showed that the response current of the electrode to AA and EP could still reach 95.2% and 96.3% of the initial current after 90 days, indicating that the electrode had good stability.

**Figure 9:**
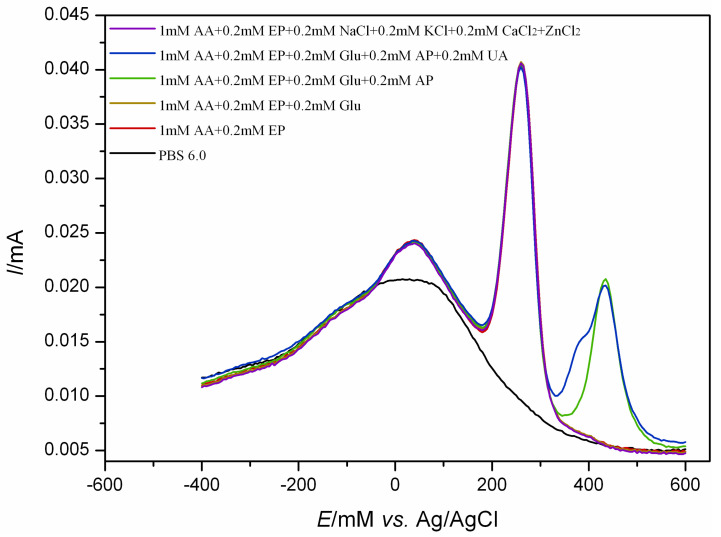
Anti-interference test of the Fe(III)-PLH-CMWCNTs/AuE against some interferents including 0.2 mM Glu, 0.2 mM AP, 0.2 mM UA, 0.2 mM NaCl, 0.2 mM KCl, 0.2 mM CaCl_2_ and 0.2 mM ZnCl_2_.

In the testing of real samples, other electrophysiological active substances such as glucose (Glu), acetaminophen (AP), uric acid (UA) and some metal ions (K^+^, Na^+^, Ca^2+^, Zn^2+^, etc.) may interfere with the detection of AA and EP. The anti-interference performance of the sensor is evaluated by detecting its electrochemical response to 1.0 mM AA and 0.2 mM EP in the presence of the above interfering substances, and the results are shown in Figure 9. As can be seen from the figure, Glu has almost no response in the scanning range of −400~600 mV, which does not interfere with the sensor detection application. UA and AP had an oxidation peak around 400 mV and 420 mV, respectively, but AA and AP had almost no effect on the response. The sensor also shows good anti-jamming performance against common metal ions in some physiological samples.

### 2.6. Analysis in Real Samples

In this study, the applicability of the constructed sensor was investigated in the detection of real samples. AA samples were extracted from commercial vitamin tablets (0.1 g/tablet) with 0.1 M PBS (pH 6.0) solution grinding at a 1:1000 mass ratio. EP samples were obtained from the diluent of medical injection (content 1 mg/branch) and diluted with 0.1 M PBS (pH 6.0) solution at a 1:1000 volume ratio. The serum samples were diluted to 1% with 0.1 M PBS (pH 6.0) solution and centrifuged at 12,000 rpm for 30 min, and the supernatant was filtered using a 0.2 μm microporous filter membrane in three stages, stored at 4 °C and placed at room temperature before use. The test results of recoveries are shown in Table 2. The recoveries of AA and EP ranged from 96.38 to 106.84% and 94.93 to 104.45 %, respectively. The total standard deviations were 2.45~5.12%. The above data show that the sensor constructed in this study has practical applicability, and they provide a certain reference value for the daily detection and clinical diagnosis of AA and EP.

## 3. Materials and Methods

### 3.1. Chemicals and Reagents

Epinephrine (EP), C_9_H_13_NO_3_ HCl, poly-L-histidine (PLH), ascorbic acid (AA), ammonium persulfate (APE), N-(3-dimethylaminopropyl)-N′-ethylcarbodiimide hydrochloride, EDC, N-hydroxysuccinimide, FeCl_3_, CoCl_2_, NiCl_2_, and CuCl_2_·2H_2_O were obtained from Sigma Aldrich (Burlington, MA, USA). Multi-walled carbon nanotubes (MWCNTs), diameter 20~50 nm, length 0.5~5 μm, purity 95%) were obtained from Chengdu Organic Chemistry Co., LTD., Chengdu, China, Chinese Academy of Sciences. Sodium borohydride, hydrogen peroxide, Na_2_HPO_4_·12H_2_O, and K_3_FeC_6_N_6_ were supplied by the Tianjin DAMAO chemical reagent factory, Tianjin, China. Pyridine and sodium borohydride were obtained from the Aladdin Industril Corporation, Beijing, China. Human blood serum was obtained from the Beijing Solarbio technology Co., LTD. (Beijing, China). In this work, all the other chemicals (99%, Merck, Darmstadt, Germany) were analytical reagents. Millipore milli-Q ultrapure water was used during the experiments.

### 3.2. Apparatus and Measurements

Talos F200X instrument (FEI, Hillsboro, OR, USA) was used for transmission electron microscopy (TEM) and energy-dispersive X-ray spectroscopy (EDX). We used a TENSOR 37 FT-IR (BRUKER, Ettlingen and Leipzig, Germany) to obtain the FT-IR spectra. The working electrode (Au electrode; φ = 3 mm), counter electrode (Ag/AgCl; saturated KCl), and reference electrode (platinum wire; φ = 1 mm) constitute a conventional three-electrode system. The 283 Potentiostat-Galvanostat electrochemical workstation (EG&G PARC with M270 software, Microsoft Windows XP) provided all the electrochemical experiments. We used a J26XP (Beckman Coulter, Brea, CA, USA) high-speed refrigerated centrifuge for cryogenic centrifuge.

### 3.3. Preparation of Carboxylated Multi-Wall Carbon Nanotubes

We weighed out 10 mg of multi-walled carbon nanotubes and added them to 40 mL of a mixed acid solution (sulfuric acid/nitric acid = 3:1, *v*:*v*) at 50 °C. This was allowed to react for 4 h under ultrasonic conditions. After the reaction was complete, it was centrifuged at 12,000 rpm for 30 min at 4 °C. The precipitate was washed with deionized water, and the centrifugation process was repeated until the pH of the solution was close to neutral. The precipitate was dried at 85 °C under vacuum, producing the carboxylated multi-walled carbon nanotubes, which were denoted as CMWCNTs.

### 3.4. Preparation of PLH Functionalized CMWCNs

Then, a 0.1 M PBS (pH 6.0) suspension of CMWCNTs of 1 mL and 1 mg mL^−1^ was prepared and fully dispersed after ultrasonic treatment. Next, 2 mM EDC (the final reaction concentration is not specified below) and 5 mM NHS were added and reacted for 15 min at room temperature. At the end of the reaction, the EDC and NHS were quenched by adding 20 mM mercaptoethanol and centrifugally suspended on 1 mL 0.1 M PBS (pH 7.0) to obtain carboxyl-activated CMWCNTs.

Next, 1 mL and 1 mg mL^−1^ PLH 0.1 M HCl solution was prepared and added to the carboxyl-activated CMWCNTs suspension, reacted for 2 h under ultrasonic conditions, and centrifugally suspended in 1 mL of deionized water to obtain PLH-functionalized CMWCNTs.

### 3.5. Construction of the Sensing Interface of Me-PLH-CMWCNTs/AuE

A gold electrode was polished into a smooth mirror by using nanometer aluminum oxide powder with a diameter of 0.3 μm, 0.1 μm and 0.05 μm successively, and soaked in Piranha solution (30% HNO_3_:98% H_2_SO_4_ = 1:3, *v*:*v*) at 90 °C for 10 min. Then, it was ultrasonically cleaned in deionized water and anhydrous ethanol alternately, 3 times for 2 min each time, and dried with high-purity nitrogen for use.

Then, 1 mL and 0.1 M of FeCl_3_, CoCl_2_, NiCl_2_, CuCl_2_, ZnCl_2_, MnCl_2_, CdCl_2_ and PdCl_2_ solutions were prepared individually. Then, 100 μL of each solution was added into a PLH-functionalized CMWCNT suspension with equal volume, and an ultrasonic reaction was performed for 3 h. After being centrifugally suspended in 100 μL deionized water, nanocomposites containing metal ions were obtained, which were denoted as Me-PLh-CMWCNTs (Me = Fe(III), Co(II), Ni(II), Cu(II), Zn(II), Mn(II), Cd(II), and Pd(II)).

**Figure 10:**
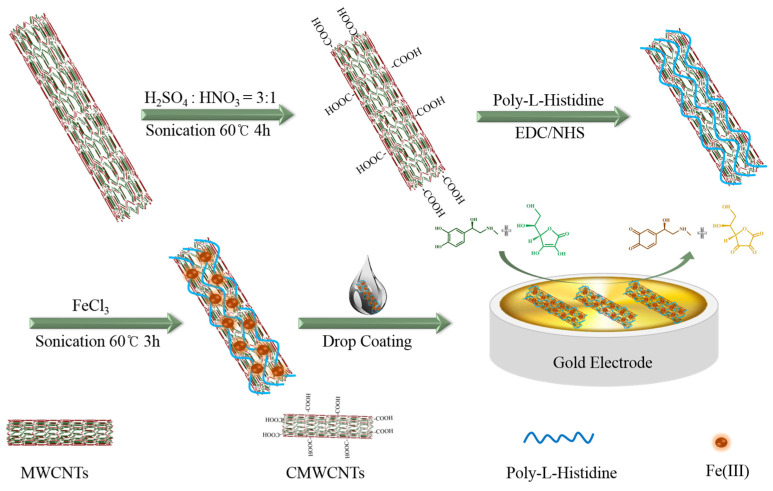
Illustration for the preparation of Fe(III)-PLH-MWCNTs/AuE.

We took 10 μL of each metal–organic nanocomposite and applied it to the surface of the newly cleaned gold electrode, dried it at room temperature, and then used it for Me-PLH-CMWCNTs/AuE (Me = Fe (III), Co (II), Ni (II), Cu (II), zinc (II), Mn (II), Cd (II), and Pd (II)). The preparation process of Fe(III)-PLH-CMWCNTs/AuE is shown in Figure 10.

### 3.6. Preparation of the Electrochemical System

Using a conventional three-electrode test system, Me-PLH-CMWCNTs/AuE was used as the working electrode, while a platinum wire (φ = 1 mm) was used as the counter electrode, and a saturated KCl-filled Ag/AgCl glass electrode was used as the reference electrode. The electrochemical catalytic performance of the prepared electrodes for epinephrine and ascorbic acid in a 0.1 M PBS system was investigated using cyclic voltammetry, differential pulse voltammetry, and other electrochemical analysis methods. All electrochemical tests were conducted at room temperature.

## 4. Conclusions

In this study, polyhistidine, as a metal ligand, modified carboxylated multi-walled carbon nanotubes and further chelated iron ions, and it innovatively synthesized a metal–organic nanocomposite with high catalytic activity of both ascorbic acid and epinephrine. To our knowledge, this strategy is the first of its kind in the construction of biosensing interfaces. Polyhistidine is a multifunctional polyelectrolyte of polypeptides. Its side-chain functional group, imidazolyl, gives the polymer special conductivity and metal-chelation ability. By combining multi-walled carbon nanotubes, the electron transfer efficiency of the biosensing interface is greatly improved, and the effective active area of the sensing interface is significantly increased. Thus, the sensing interface can absorb more detection substrates, which fundamentally determines that the constructed sensor has higher sensitivity, a wider detection range, and a lower detection limit. The sensor also has good repeatability, stability, and anti-interference performance, and it has practical application value in real sample detection.

## Figures and Tables

**Table 1:** Comparison of analytical parameters between Fe(III)-PLH-CMWCNTs/AuE and the sensors previously reported by others.

Electrode Material	AA			EP			Ref.
	LOD (μM)	Liner Range (mM)	Sensitivity (μA mM^−1^)	LOD (μM)	Liner Range	Sensitivity (μA mM^−1^)	
PSAF-MCPE ^a^	0.47	0.1–0.45	-	0.61	100–350 μM	-	[29]
molybdenum disulfide quantum dots	0.05	0.005–0.04	-	0.2	0.2–40 μM	-	[30]
GCE/MWCNTs-CH ^b^	16.3	0.0531–2.32	10	3.92	22.5–547 μM	68.7	[31]
PXSP ^c^/GCE	4.0	0.01–1.343	9	0.1	2–390 μM	99	[32]
Ox-PAP ^d^/GCE	9.5	0.006–0.07	4.3	0.009	0.4–8.0 μM	279.3	[33]
Fe(III)-PLH-CMWCNTs/AuE	0.1	0.001~5.0	4.18	0.01	0.2–100 μΜ; 0.1–1.0 mM	265.75; 50.98	This Job

^a^ poly(safranin)-modified carbon paste electrode (MCPE); ^b^ chitosan; ^c^ P-xylenolsulfonephthalein; ^d^ over-oxidized poly(p-aminophenol).

**Table 2:** Determination of AA and EP in real-life samples by the prepared biosensor.

Sample	HPLC Detected (μM)	Present Method (μM)	RSD (%)	AA and EP Added (μM)	AA and EP Found (μM)	Recovery (%)	RSD (%)
AA sample 1	49.67	51.01	2.57	AA: 100 EP: 10	AA: 154.43 EP: 9.76	AA: 103.42 EP: 97.60	3.45 4.78
AA sample 2	53.32	54.62	3.64	AA: 200 EP: 20	AA: 257.86 EP: 19.09	AA: 101.62 EP: 95.45	3.54 4.92
AA sample 3	62.18	63.35	4.53	AA: 300 EP: 30	AA: 352.49 EP: 31.33	AA: 96.38 EP: 104.43	3.21 3.84
EP sample 1	4.96	4.71	3.78	AA: 100 EP: 10	AA: 97.47 EP: 15.12	AA: 97.47 EP: 104.10	3.87 4.28
EP sample 2	5.12	4.85	5.12	AA: 200 EP: 20	AA: 201.45 EP: 25.74	AA: 100.72 EP: 104.45	3.93 4.82
EP sample 3	5.47	5.87	4.72	AA: 300 EP: 30	AA: 307.39 EP: 34.35	AA: 102.46 EP: 94.93	2.45 3.12
Serum sample 1	-	-	-	AA: 100 EP: 10	AA: 97.68 EP: 10.14	AA: 97.68 EP: 101.40	4.43 3.67
Serum sample 2	-	-	-	AA: 200 EP: 20	AA: 213.67 EP: 19.84	AA: 106.84 EP: 99.20	4.43 3.11
Serum sample 3	-	-	-	AA: 300 EP: 30	AA: 303.48 EP: 29.29	AA: 101.60 EP: 97.63	3.45 2.89

## Data Availability

The original contributions presented in the study are included in the article/supplementary materials, further inquiries can be directed to the corresponding author/s.

## Supplementary Materials

The supporting information can be downloaded at: https://www.mdpi.com/article/10.3390/ijms25147883/s1.

## Abbreviations

EP	Epinephrine
AA	Ascorbic acid
CNTs	carbon nanotubes
CMWCNTs	Carboxylated multi-walled carbon nanotubes
PLH	Poly-L-histidine
APE	Ammonium persulfate
TEM	Transmission electron microscopy
EDX	Energy-dispersive X-ray spectroscopy
HADDF	high-angle toroidal dark field
Glu	Glucose
AP	Acetaminophen
UA	Uric acid
XPS	X-ray photoelectron spectroscopy
FTIR	Fourier transform infrared spectrometer
