# Supplementary figures and images for: A Biosensor for Simultaneous Detection of Epinephrine and Ascorbic Acid Based on Fe(III)–Polyhistidine-Functionalized Multi-Wall Carbon Nanotube Composites
Source: Int J Mol Sci. 2024 Jul 18;25(14):7883. doi: 10.3390/ijms25147883 (PMC11276898; doi:10.3390/ijms25147883)

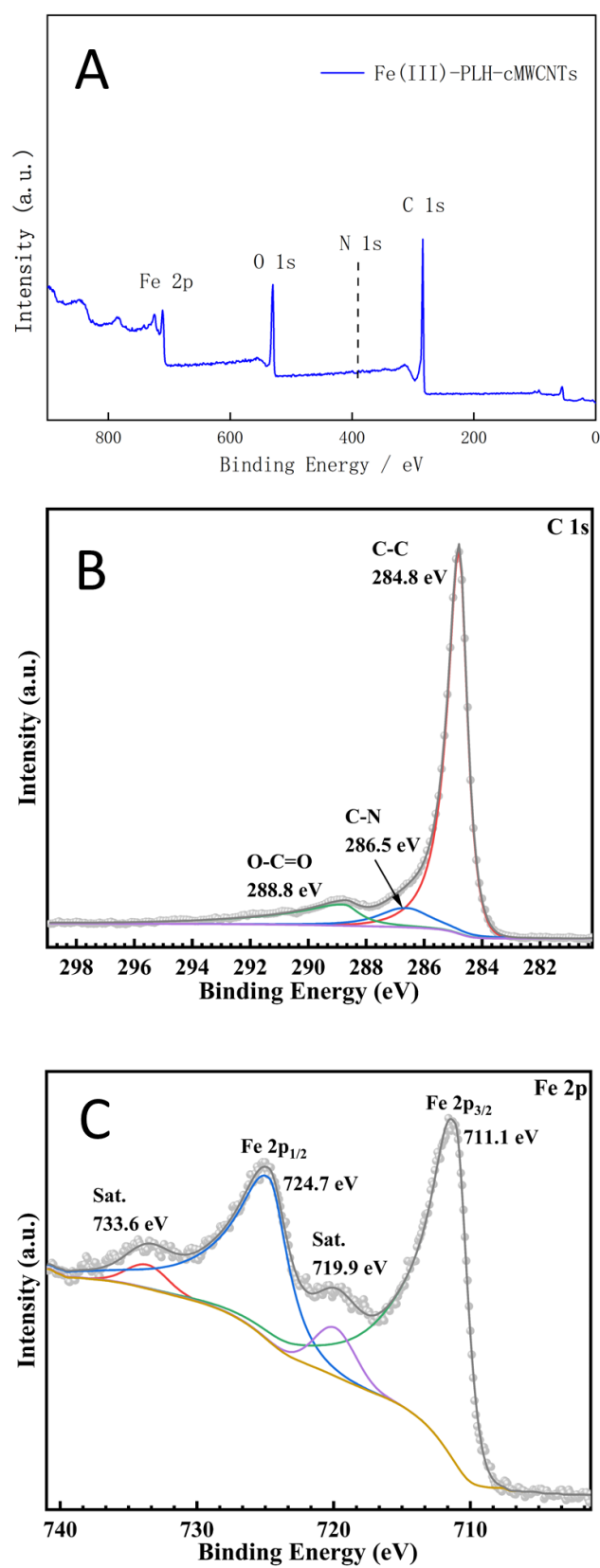

**Figure S1.** The XPS spectrum of Fe(III)-PLH-CMWCNTs (A), C 1s (B) and Fe 2p (C).

Supplement: Supplementary file 1 [file ijms-25-07883-s001.zip › ijms-3105438-supplementary.pdf]
